# A systematic review to explore patients’ MS knowledge and MS risk knowledge

**DOI:** 10.1007/s10072-024-07541-5

**Published:** 2024-05-03

**Authors:** Edward Smith, Dawn Langdon

**Affiliations:** Department of Psychology, Royal Holloway University of London, Egham, UK

**Keywords:** Multiple sclerosis, Multiple sclerosis knowledge, Multiple sclerosis risk knowledge, Patient education

## Abstract

Living with a chronic illness poses particular challenges, including maintaining current disease knowledge to optimise self-management and interaction with health professionals. People with Multiple Sclerosis (MS) are increasingly encouraged to participate in shared decision making. Making informed decisions is likely to rely on adequate knowledge about the condition and its associated risks. The aim of this systematic review is to explore patients’ existing MS knowledge and MS risk knowledge, and how these relate to demographic and disease variables. A literature search was conducted using PsycINFO, PubMed and Cochrane Library. Eligible studies were published peer-reviewed reporting quantitative measures of MS knowledge and MS risk knowledge in adult MS patients. Eighteen studies met inclusion criteria comprising a total sample of 4,420 patients. A narrative synthesis was undertaken because studies employed various measures. Suboptimal levels of MS knowledge and MS risk knowledge were generally identified across studies. Greater self-reported adherence and a willingness to take medication were related to higher MS knowledge, while educational level was a significant predictor of both MS knowledge and MS risk knowledge. Associations with other demographic and disease-related variables were mixed for both knowledge domains. Direct comparison of results across studies were limited by methodological, sampling and contextual heterogeneity. The review’s findings and implications for future research and clinical practice are considered from this perspective.

**Supplementary Information:**

The online version contains supplementary material available at 10.1007/s10072-024-07541-5.

## Introduction

Multiple Sclerosis (MS) is a chronic inflammatory disease of the central nervous system (CNS) resulting in neurodegeneration and neurological disability [1]. Primarily starting in young adulthood, it is estimated 2.3 million people live with MS globally, with MS being more prevalent in women. The course of MS can be unpredictable, with prognosis varying between individuals. A constellation of symptoms are associated with MS, across the physical and psychological domains, including sensory and motor impairments, fatigue and cognitive and mood difficulties. Disease modifying drugs (DMDs) can delay the progression of MS. Nine different drug classes with more than a dozen approved therapies are now available [2], presenting a range of benefit and risk profiles, with more effective treatments carrying greater risk of severe side-effects [3]. This has created a complex information landscape for MS patients to assimilate [4]. Adherence to DMDs is commonly suboptimal [5] and is multifactorially determined [6], including risk attitude [7]. Consequently, clinical management of MS is complex.

Involving patients in shared treatment decisions has been recommended [8]. Shared decision making is likely to be underpinned by a person’s knowledge of MS and their MS risk knowledge [9, 10]. These represent arguably separable types of health knowledge. MS knowledge represents a more general understanding of MS, such as its aetiology, assessment, diagnosis, incidence, prevalence, pathology and treatment [11]. MS risk knowledge is a focused and evidence-based understanding of specific risks associated with treatments and disease progression, such as accumulation of disability, efficacy and risks of treatments, accuracy of diagnostic procedures, and recognising uncertainties in the disease course [12]. The risk and benefit profile of the many licensed DMDs is a complex information landscape, which was further complicated by the arrival of COVID-19 [13, 14].

Measures exist for MS knowledge and MS risk knowledge. Giordano et al. [11] developed and validated the Multiple Sclerosis Knowledge Questionnaire (MSKQ) which is a twenty-five-item self-report questionnaire exploring multiple facets of MS knowledge. To measure MS risk knowledge, Heesen et al. [15] developed the MS risk knowledge questionnaire (MSK, also referred to as the RIKNO), which was later adapted by Heesen et al. [16] in their development of the Risk Knowledge Questionnaire 1.0 (RIKNO 1.0). The measure was further revised by Heesen et al. [10] in their development of the Risk Knowledge Questionnaire 2.0 (RIKNO 2.0). MS knowledge and MS risk knowledge have been treated as independent constructs in research. Studies using measures of both knowledge domains have identified small to moderate correlations between patients’ scores [10, 11, 17].

Despite the importance of knowledge in shared decision making, patients have reported unmet needs in the provision of education and peer support [15, 18, 19]. MS patient advocates and health professionals have also argued for increased education and collaborative engagement in healthcare [20]. This would likely deliver health initiatives which advocate patient involvement and informed decision making. MS knowledge can be modified through provision of evidence-based MS information [21]. However, methodological differences in the delivery of interventions and measurement of knowledge have precluded definitive conclusions about their comparative effectiveness. MS risk knowledge has implications for disease management by supporting patients to make decisions about their treatment soon after diagnosis, reducing risk of unrealistic treatment expectations undermining treatment adherence [17]. Studies have identified deficiencies in patients’ MS risk knowledge, which may compromise health decision making and outcomes [22]. Fortunately, MS risk knowledge can be enhanced through educational interventions [23].

Understanding patients’ knowledge characteristics and factors associated with these is important. It would be helpful to characterise the nature and degree of patients’ MS knowledge and MS risk knowledge. Consideration of patient demographics and disease-related factors (for example, DMD use) may also elucidate how these relate to patients’ knowledge and information needs, which could inform tailored interventions. This review seeks to explore these factors whilst addressing the following questions:

1. What is the nature and degree of patients’ MS knowledge?
2. What is the nature and degree of patients’ MS risk knowledge?
3. Are there demographic or disease-related factors which relate to MS knowledge?
4. Are there demographic or disease-related factors which relate to MS risk knowledge?

To our knowledge, this is the first systematic review evaluating both MS knowledge and MS risk knowledge alongside demographic and disease-related factors.

## Materials and methods

The Preferred Reporting Items for Systematic Reviews and Meta-Analyses (PRISMA) [24] was used for conducting and presenting the systematic review.

### Search strategy

**Fig. 1:**
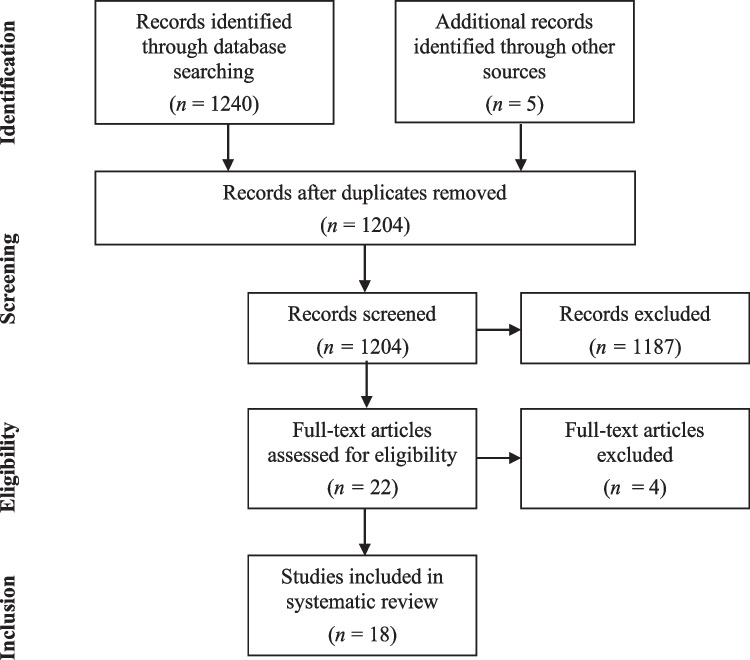
PRISMA chart for study selection process

Relevant search terms were entered into three electronic databases (PsycINFO, PubMed and Cochrane Library) on 8 November 2020. The following search terms were used: “(multiple AND sclerosis)” AND “(multiple AND sclerosis AND knowledge)” OR “(multiple AND sclerosis AND understanding)” OR “(multiple AND sclerosis AND comprehension)” OR “(multiple AND sclerosis AND risk knowledge)” OR “(multiple AND sclerosis AND risk comprehension)” OR “(multiple AND sclerosis AND risk understanding)”. The identified 1,240 titles and abstracts were screened by one researcher (ES) according to the inclusion and exclusion criteria to determine their suitability for full-text review. Suitable full-text articles were then reviewed by one reviewer (ES). Reference lists of full-text articles were checked to identify further relevant studies for review. Full-texts for inclusion were sent to another researcher (DL) for review which preceded a discussion between the researchers about their suitability for inclusion. Figure 1 outlines the search strategy.

### Eligibility criteria

Published peer-reviewed articles reporting on baseline measures of MS knowledge and MS risk knowledge were included if they were quantitative, utilised experimental, correlational or differential research methods and included adult participants with any MS subtype. Studies which employed differential research methods included those which compared knowledge according to preexisting patient characteristics (e.g. MS subtype). Articles were excluded if they were review papers, not written in English, exclusively qualitative or included participants below the age of eighteen years or with clinical conditions other than MS.

### Data extraction

Extracted data included participant recruitment, methodological details, baseline demographics, baseline disease-related data, and baseline MS knowledge and MS risk knowledge data. Study demographics and results are summarised in Supplementary Information 1 (Table S1) and Supplementary Information 2 (Table S2), respectively.

## Results

Eighteen studies met inclusion criteria and comprised a total sample of 4,420 patients. This included 2,883 (68.9%) patients with relapsing–remitting MS (RRMS), 407 (9.7%) patients with secondary-progressive MS (SPMS), 279 (6.7%) patients with primary progressive MS (PPMS), 178 (4.3%) patients defined as early RRMS (including Clinically Isolated Syndrome, which not all countries routinely document), and 33 (0.8%) patients with progressive-relapsing MS (PRMS). A further 402 (9.61%) patients had an ‘unclear’ diagnosis. One study did not provide information on patient diagnoses [9], with diagnostic information available for 4,182 patients. Gender information was available for 4,320 patients. The mean age of patients was 40.6 years and 3,013 (65.3%) patients were female. Country of recruitment was reported in 17 studies, with 1,979 (45.8%) recruited from Germany, 995 (23%) from Italy, 742 (17.2%) from the USA, 200 (4.6%) from Saudi Arabia, 152 (3.5%) from Australia, 96 (2.2%) from Brazil, 81 (1.9%) from Canada, 29 (0.7%) from Spain, 20 (0.5%) from Turkey, 14 (0.3%) from the Netherlands and 11 (0.3%) from Serbia.

### Quality assessment

The EPHPP Quality Assessment Tool for Quantitative Studies [25] was used to assess study quality (Table 1). Fourteen studies were rated overall ‘weak’, 1 overall ‘moderate’ and 3 overall ‘strong’. Data from studies with ‘weak’ ratings should be considered cautiously due to having less control over additional factors potentially implicated in the results than studies with randomised-controlled designs.

**Table 1:** Study quality ratings

Author	Selection Bias	Design	Confounders	Blinding	Data Collection	Withdrawals and Dropouts	Overall Rating
Abulaban et al. [9]	Weak	Weak	Weak	Moderate	Strong	NA	Weak
Bichuetti et al. [37]	Moderate	Weak	Weak	Moderate	Weak	NA	Weak
Bruce et al. [26]	Moderate	Weak	Weak	Moderate	Weak	NA	Weak
Bruce et al. [27]	Moderate	Weak	Weak	Moderate	Moderate	NA	Weak
Feicke et al. [31]	Moderate	Strong	Strong	Moderate	Weak	Strong	Moderate
Giordano et al. [11]	Moderate	Weak	Weak	Moderate	Strong	NA	Weak
Giordano et al. [28]	Moderate	Weak	Weak	Moderate	Strong	NA	Weak
Heesen et al. [15]	Moderate	Weak	Weak	Moderate	Weak	NA	Weak
Heesen et al. [16]	Moderate	Weak	Weak	Moderate	Strong	NA	Weak
Heesen et al. [10]	Weak	Weak	Strong	Moderate	Strong	NA	Weak
Heesen et al. [38]	Moderate	Moderate	Weak	Moderate	Weak	Weak	Weak
Hofmann et al. [39]	Weak	Moderate	Weak	Moderate	Weak	NA	Weak
Jarmolowicz et al. [29]	Moderate	Weak	Weak	Moderate	Weak	NA	Weak
Köpke et al. [34]	Moderate	Strong	Strong	Strong	Moderate	Strong	Strong
Köpke et al. [35]	Moderate	Strong	Strong	Moderate	Moderate	Strong	Strong
Prunty et al. [32]	Weak	Strong	Strong	Moderate	Weak	Moderate	Weak
Rahn et al. [36]	Moderate	Strong	Strong	Moderate	Moderate	Moderate	Strong
Skinner et al. [33]	Moderate	Weak	Weak	Moderate	Weak	NA	Weak

### MS knowledge

#### Validated measures

Seven studies used validated MS knowledge measures. Abulaban et al. [9] conducted an internet survey to assess patients’ MS knowledge in Saudi Arabia. A large sample of MS patients completed the MS Knowledge Questionnaire (MSKQ) [11]. With a mean score of 13.6 (SD = 3.6) out of a maximum of 23, the authors concluded patients’ knowledge of MS disease types and treatment was lower relative to their knowledge of its pathophysiology.

Bruce et al. [26] assessed how MS patients weigh disease modifying therapy (DMT) risks and benefits when making treatment decisions. Patients completed a medical decision making task to assess their willingness to take a hypothetical DMT as their efficacy and side effects probabilities varied. Patients completed the MSKQ to assess how MS knowledge related to self-reported likelihood of initiating treatment. The authors reported a mean MSKQ score of 17.4 (SD = 3.4).

Using an analogous medical decision making task, Bruce et al. [27] investigated how patients weigh treatment decisions, with specific consideration given to how patients discount DMT efficacies and side effects. The association of demographics, clinical characteristics, MS knowledge, cognitive and emotional functioning, and treatment adherence were studied. The authors obtained a mean MSKQ score of 17.5 (SD = 3.4). The authors further observed poorer cognitive functioning was related to greater benefit discounting.

To measure MS knowledge, Giordano et al. [11] developed and validated the MSKQ on a small sample of MS patients. Further to compiling the final 25-item multiple choice questionnaire, newly diagnosed patients completed the MSKQ, with a median score of 17 obtained from a possible score of 25.

Giordano et al. [28] measured cross-cultural differences in MS knowledge and MS risk knowledge using the MSKQ and Risk Knowledge questionnaire 2.0 (RIKNO 2.0) [10]. The mean MSKQ score was 19.3 (SD = 3.2; *n* = 298).

In their validation of the RIKNO 2.0, Heesen et al. [10] invited a large group of MS patients to complete the MSKQ and RIKNO 2.0. Measures of patient demographics, clinical characteristics, disease severity, quality of life and self-reported cognitive functioning were collected. Heesen et al. [10] obtained a mean MSKQ score of 20.1 (SD = 2.6).

Examining MS patients’ sensitivity to DMT prices and how these related to adherence and MS knowledge, Jarmolowicz et al. [29] invited a large sample to complete the MSKQ and measures of demographics, disease severity, emotional and cognitive functioning, adherence and a medication purchasing task. The mean MSKQ score obtained was 17.1 (SD = 3.5). A recent review of MS disease-related knowledge measures has only provisionally recommended use of the MSKQ and RIKNO, until further psychometric evidence is provided [30].

#### Unvalidated measures

Four studies used bespoke knowledge measures within intervention studies to assess baseline MS knowledge. Feicke et al. [31] compared the effectiveness of a self-management training programme with a standard information brochure. Self-management ability and MS knowledge were measured before, immediately post- and 6 months post-intervention. Patients showed a good degree of MS knowledge at baseline, with more than 75% correct responses provided.

Patients’ perceived knowledge of therapeutic decisions was investigated by Heesen et al. [15]. A large sample of MS patients completed questionnaires of their self-reported knowledge, information interests and treatment decisional role preferences. Most patients rated their subjective perceived level of knowledge as 63% (100% representing maximal subjective knowledge).

Evaluating a decision aid for female MS patients considering beginning or enlarging their families, Prunty et al. [32] measured changes in MS knowledge, decisional self-efficacy, and decisional conflict. Patients completed baseline knowledge measures in relation to the decisional aid. From a maximum score of 10, mean knowledge scores did not significantly differ between those who then received the intervention (4.1) or control (4.2) condition.

Skinner et al. [33] studied expectations for receiving a genetic counselling session in which family-specific recurrence risks were discussed in a small sample of MS patients. 43.5% reported having an ‘average’ understanding of MS aetiology, 17.8% reported a ‘greater than average’ and 38.7% reported a ‘less than average’ understanding.

### Factors associated with MS knowledge

Studies have measured demographic or disease-related variables alongside MS knowledge to examine their association.

#### Age

Assessing correlates of MS knowledge, Giordano et al. [11] found MS knowledge scores were not associated with age (Odds Ratio (OR): 1.1; Confidence Interval (CI): 0.5–2.2; *p* = 0.27). Similarly, Heesen et al. [10] found MS knowledge was not associated with age (β = 0.03, *p* = 0.486). Generally, age has not been associated with MS knowledge.

#### Gender

Giordano et al. [11] studied the association between female gender and MSKQ scores. A positive correlation was identified between the two factors (OR: 2.2; CI: 1.0–4.6; *p* = 0.03). Abulaban et al. [9] found MSKQ scores were marginally higher in female patients. However, Heesen et al. [10] did not find a strong relationship between gender and MSKQ scores (β = 0.03, *p* = 0.442). Studies have yielded mixed results with regards to gender and MS knowledge.

#### Education

Giordano et al. [11] found MSKQ scores positively and significantly correlated with higher educational attainment (OR: 5.0; CI: 1.7–14.4; *p* = 0.001). Heesen et al. [10] also found higher levels of MS knowledge correlated significantly with higher levels of education (β = 0.26, *p* < 0.001). Further, Abulaban et al. [9] found higher MSKQ scores positively correlated with higher levels of educational attainment (*p* = 0.07). Conversely, Skinner et al. [33] found the percentage of correct knowledge scores did not differ according to educational level (*p* = 0.915). Apart from one study, positive relationships between higher educational level and MS knowledge have been identified.

#### Ethnicity

Giordano et al. [28] compared MSKQ scores between patients from 3 countries*.* MSKQ scores in Germany (*n* = 117; mean = 19.8; SD = 3.1), Italy (*n* = 53; mean = 18.3; SD = 3.6) and the Netherlands (*n* = 128; mean = 19.1; SD = 3.1) were similar. No further studies measured ethnicity as a correlate of MS knowledge.

#### Disease duration

Giordano et al. [11] found higher MSKQ scores significantly correlated with a shorter disease duration (*p* = 0.03). Conversely, Skinner et al. [33] found MS knowledge scores did not differ according to time since diagnosis (*p* = 0.31). Relying on a scattering of studies, results have been mixed in relation to disease duration.

#### DMDs

Bruce et al. [26] found higher MSKQ scores positively correlated with increased willingness to take DMDs (ρ = 0.28, *p* < 0.001). In relation to self-reported treatment adherence determination, Bruce et al. [27] reported a significant correlation with higher MS knowledge (*r* = 0.22, *p* = 0.002). Jarmolowicz et al. [29] reported lower level of price sensitivity to DMDs correlated with higher MS knowledge (*r* = -0.24, *p* < 0.01). Varied factors related to DMD use have been associated with higher MS knowledge, including willingness to take medication and self-reported medication adherence.

#### Disability

Heesen et al. [10] found MSKQ scores were not associated with self-reported disability (β =  − 0.02, *p* = 0.595). No further studies measured disability as a correlate of MS knowledge.

#### Mood

Giordano et al. [11] found MS knowledge was not associated with anxiety (OR: 0.6; CI: 0.3–1.3; *p* = 0.16). Studies correlating mood with MS knowledge have been limited, whilst no studies measured the association with MS risk knowledge.

#### Cognition

In relation to cognition, Heesen et al. [10] found self-rated cognitive functioning was not significantly correlated with MSKQ scores (β =  − 0.07, *p* = 0.073) or RIKN0 2.0 scores (β =  − 0.07, *p* = 0.052). No further studies measured the association between mood and MS knowledge or MS risk knowledge.

### MS risk knowledge

#### Validated measures

Seven studies used validated MS risk knowledge measures within survey designs or as baseline measures in intervention studies.

Giordano et al. [28] summarised patients’ risk knowledge scores on the RIKNO 2.0. From a possible score of 21, the mean RIKNO 2.0 score was 8.7 (SD = 3.5; *n* = 1153). MSKQ scores were significantly higher than RIKNO 2.0 scores (adjusted mean difference = 36.2; *p* < 0.01).

Alongside investigating MS patients’ perceived level of knowledge, information interests and decisional role preferences, Heesen et al. [15] developed the MSK to assess MS risk knowledge. From a maximum score of 19, the authors described patients having low risk knowledge, with a mean score of 6.4 (SD = 2.4) obtained.

Heesen et al. [16] developed the RIKNO 1.0 to assess MS risk knowledge. In a pilot study, a small group of patients received 4 sessions of an educational programme and completed the questionnaire. The programme entailed a 4-h educational session on MS diagnosis, prognosis and treatment. The mean baseline RIKNO 1.0 score was 9.8 (SD = 3.2).

In validating the RIKNO 2.0, Heesen et al. [10] obtained a mean RIKNO 2.0 score of 8.9 (SD = 3.6) from a large sample. The authors observed higher scores were generally obtained on the MSKQ.

Köpke et al. [34] compared the efficacy of an educational programme with a stress management control condition in promoting informed treatment decision making and patient autonomy. Risk knowledge was measured using the MSK at baseline and 2 weeks post-intervention. ‘Good risk knowledge’ was classed as attaining at least a score of 12 out of 19. At baseline, the mean MSK score was 10.6 (SD = 2.6) for those who then received the intervention and 9.4 (SD = 2.9) for those in the control condition.

Similarly, Köpke et al. [35] compared the effectiveness of a 6-h educational programme with standard information in promoting informed decision making. Adequate risk knowledge was defined as at least 9 correct responses out of 19 on the RIKNO 1.0. At baseline, the mean score was 6.1 (SD = 2.8) for those who then received the intervention and 6.51 (SD = 2.5) for those in the control condition.

In an RCT, Rahn et al. [36] compared the efficacy of a decision coaching programme on DMD benefits and risks with care as usual in a small group of patients with RRMS. From a maximum score of 19, mean baseline RIKNO 1.0 scores were 8.3 (SD = 3.4) for intervention group and 8.1 (SD = 3.1) for control group patients.

#### Unvalidated measures

Three studies used bespoke MS risk knowledge measures within survey or intervention studies. Bichuetti et al. [37] measured knowledge of risks associated with Natalizumab (NAT) in a small sample of Brazilian patients with RRMS. Patients considered MS a severe disease, and generally understood risks associated with NAT, with most patients considering risk of PML as ‘moderate to high’.

Heesen et al. [38] investigated patients’ understanding and acceptance of risks associated with NAT, including risk of PML. The authors reported on data from two trials (PERCEPT and CONSIDER). In the CONSIDER trial, data were collected from a subset of PERCEPT trial patients at baseline, 1 month and at 12 month follow-up regarding knowledge of NAT efficacy and side effects. 61.6% of patients were aware PML is unlikely in the first year of treatment and 64.6% could identify outcomes associated with having all three risk factors present. 51.5% underestimated the risk of PML following 2 years of therapy. 29.3% provided a correct response regarding degree of risk increase due to deficient immunosuppression.

In assessing MS patients’ knowledge of risks associated with taking Mitoxantrone (MITOX), Hofmann et al. [39] invited patients to complete a questionnaire before and after they received evidence-based information. At baseline, 40% of patients correctly selected the correct risk for leukaemia and 16% selected the correct risk for cardiotoxicity. 58% underestimated risks for leukaemia and 82% underestimated risks of cardiotoxicity.

### Factors associated with MS risk knowledge

Alongside measuring MS risk knowledge, studies have measured associations between knowledge and demographic or disease-related variables.

#### Age

Heesen et al. [16] found younger age correlated with higher RIKNO 1.0 scores (β = - 0.22, *p* = 0.002) in patients who completed the PEDAPIP trial [34]. Similarly, Heesen et al. [15] found age negatively correlated with higher MSK scores (*r* = - 0.46, *p* < 0.001). However, in validating the RIKNO 2.0, Heesen et al. [10] found MS risk knowledge was not associated with age (β =  − 0.06, *p* = 0.093). Similarly, Heesen et al. [38] found age did not predict knowledge of PML risk when commencing NAT in patients with RRMS. Hofmann et al. [39] found patients’ knowledge of risks associated with taking MITOX did not differ according to patients’ age. In summary, the association between age and knowledge has generally been inconsistent.

#### Gender

Heesen et al. [10] found RIKNO 2.0 scores were not associated with female gender (β =  − 0.02, *p* = 0.558). Heesen et al. [38] found gender was not predictive of risk knowledge for developing PML when taking NAT. Further, Hofmann et al. [39] found gender was not associated with estimation of risks for developing leukaemia when taking MITOX. Relationships between gender and knowledge have been inconsistent between studies.

#### Education

Heesen et al. [16] found higher levels of educational attainment significantly correlated with higher MS risk knowledge (β = 0.21, *p* = 0.005). Heesen et al. [10] found higher levels of education (β = 0.3, *p* < 0.001) were positively and significantly associated with higher RIKNO 2.0 scores. Relationships between education and risk knowledge have been consistent.

#### Ethnicity

Giordano et al. [28] noted higher mean scores were obtained in Germany (*n* = 242; mean = 9.3; SD = 4.4) and Serbia (*n* = 107; mean = 11.7; SD = 3.6), whilst scores in Italy (*n* = 100; mean = 7.2; SD = 3.1), Spain (*n* = 363; mean = 6.3; SD = 3.5), Turkey (*n* = 203; mean = 6.6; SD = 2.7) and the Netherlands (*n* = 138; mean = 9; SD = 2.7) were similar.

#### MS subtype

In evaluating disease course as a determinant of MS risk knowledge, Heesen et al. [15] found mean MSK scores were highest in patients diagnosed within the previous year (mean = 8.3; SD = 3), followed by RRMS patients (mean = 7.2; SD = 2.6) and PPMS patients (mean = 5.2; SD = 2.6) (*F*(2,166) = 15.9, *p* = 0.001). Heesen et al. [16] identified a relapsing–remitting course was significantly associated with higher RIKNO 1.0 scores (β = 0.22, *p* = 0.002). A limited number of studies correlated MS subtype with MS risk knowledge, whilst no studies measured the association with MS knowledge.

#### Disease duration

Heesen et al. [38] found time since diagnosis was not predictive of risk knowledge for developing PML when receiving NAT. In evaluating how responses on the MSK differed according to years since diagnosis, Heesen et al. [15] noted recently diagnosed patients obtained the highest MSK scores. Although few studies have measured an association between disease duration and risk knowledge, studies have identified mixed relationships between these variables.

#### DMDs

Heesen et al. [16] identified an autonomous preference for making treatment decisions negatively correlated with higher RIKNO 1.0 scores (β = - 0.19, *p* = 0.01). Heesen et al. [38] found length of NAT treatment was not predictive of risk stratification knowledge for developing PML. Heesen et al. [15] found patients in receipt of interferon therapies provided more correct calculations of the therapeutic effects of their therapies (mean = 7.4; SD = 2.7) than patients not in receipt of these therapies (mean = 6.2, SD = 2.8) (*F*(1,167) = 9.5, *p* = 0.002). Hofmann et al. [39] found MS risk knowledge did not differ between patients taking DMDs over the past 5 years or earlier (*p* = 0.31). Studies associating a range of factors relating to DMDs and MS risk knowledge have produced mixed results.

#### Disability

Heesen et al. [10] found RIKNO 2.0 scores were not associated with PDDS scores (β =  − 0.07, *p* = 0.049). Further, Heesen et al. [38] found baseline EDSS scores were predictive of risk stratification knowledge for developing PML when taking NAT (β = - 0.25, *p* = 0.033).

### Summary

Generally suboptimal levels of MS knowledge and MS risk knowledge were identified across studies. Because some studies used unvalidated measures, it is difficult to compare studies. Significant positive relationships have been demonstrated between higher levels of educational attainment and greater levels of knowledge, with relationships with MS risk knowledge being consistent. Associations between greater MS knowledge and varied aspects of DMD use were observed, while relationships between both knowledge domains and other demographic and disease-related variables were inconsistent.

## Discussion

This review explored patients’ MS knowledge and MS risk knowledge, and how these related to demographic and disease-related variables. Outcomes from MS knowledge and MS risk knowledge measures were variable, with suboptimal levels of knowledge identified. Studies measuring demographic and disease-related correlates of MS knowledge reported mixed findings. Both MS knowledge and MS risk knowledge were significantly and positively associated with educational attainment, indicating inequitable access to this key health information.

The MSKQ has been validated in MS patients and was used in seven studies to assess MS knowledge. Mean scores ranged from 13.6 to 20.1. Four studies used unvalidated measures within trials to measure patients’ MS knowledge at baseline and post-interventions. Three versions of the RIKNO [15] were used in seven studies to measure MS risk knowledge. With different versions containing varied questions and maximum scores, direct comparison of scores is difficult. Seven studies used unvalidated measures of MS knowledge or MS risk knowledge. It is difficult to determine the consistency with which domains of MS knowledge or MS risk knowledge were measured across these studies. Their reliability is also unclear, due to their likely addressing the specific needs and characteristics of patients within their development location. The measures used also do not include categorical ranges to define levels of knowledge or those which are deemed sufficient to inform decision making. With regards to MS risk knowledge, researchers have defined a priori thresholds to evaluate the effectiveness of interventions. Köpke et al. [34] defined a minimum score of nine on the RIKNO 1.0 as ‘adequate’ to support informed decision making. Similarly, Köpke et al. [35] defined a minimum RIKNO 1.0 score of twelve for ‘good risk knowledge’. Applying these criteria to baseline mean RIKNO 1.0 scores, two studies reported mean scores which met criteria for ‘adequate’ risk knowledge, while one did not reach this threshold. As regards mean RIKNO 1.0 scores, none of the included studies met Köpke et al.’s [35] threshold for ‘good risk knowledge’. The absence of agreed knowledge thresholds precludes the ability to consistently summarise patients’ knowledge across studies.

Significant relationships between higher levels of educational attainment and higher MS knowledge or risk knowledge were observed. Maybury and Brewin [40] identified higher MS knowledge in patients with higher educational levels. Higher levels of education were also identified as a significant predictor of higher MS risk knowledge. Relationships between lower educational attainment and poor risk knowledge have been observed in other health conditions, including cardiovascular disease [41], cancer [42] and HIV [43]. Low health literacy has been correlated with lower levels of education [44] and may mediate the relationship between educational attainment and disease knowledge [45]. With regards to MS, health literacy may therefore have important implications for patients’ MS knowledge and MS risk knowledge, including the implementation of health decisions and behaviours [46]. An association between knowledge and willingness to take DMDs [26] and adherence to treatment [27] was established. This suggests knowledge likely has an important role in DMD use and adherence, and it follows that adherence may profit from interventions which are effective at improving patients’ knowledge.

A desire for greater information, communication about prognoses and alignment between clinicians’ and MS patients’ priorities has been reported [47]. Patients may seek to fill information gaps before clinical consultations, risking exposure to unreliable information without professional support. With the internet and social media being increasing used sources of information [48], it is important to identify and promote accurate sources of information to inform decision making. Intervention studies have highlighted how knowledge can be modified. Prunty et al. [32] observed increased MS knowledge, self-efficacy and decisional certainty in patients assigned to receive a decisional aid for starting a family or a control group condition. However, Feicke et al. [31] found a self-management training programme did not impact MS knowledge compared to an information brochure control condition. As regards MS risk knowledge, Köpke et al. [34] found a greater proportion of patients reached criteria for adequate risk knowledge after a short educational intervention than a stress-management control condition. Köpke et al. [35] further found a greater proportion of participants reached informed decision making than controls following a short educational intervention. Rahn et al. [36] found a decision coaching programme led to a greater proportion of patients reaching informed treatment choices than controls, with modest improvement in MS risk knowledge observed in both groups. Hofmann et al. [39] found patients were more likely to provide correct estimates of risks following Mitoxantrone treatment. Measurement differences between studies make it difficult to compare changes in knowledge across studies. This accords with Reen et al.’s [23] review of fifteen intervention studies in which reductions in underestimating DMD risks were observed in MS patients, although reductions in overestimating benefits were less pronounced. Recently, it has been demonstrated that educational interventions to improve MS knowledge can be successfully delivered online, with potential to reach a larger cohort of patients [49].

### Limitations

Included studies were published between 2004 and 2020; the landscape for understanding and treating MS has evolved over this time. For instance, the development and licencing of DMDs has advanced since 2004, with the addition of DMDs such as Fingolimod in 2010 and Alemtuzumab in 2014 [50]. When a new MS drug is licensed, these receive considerable coverage on MS charity websites and in the general press [51]. The timing of studies may therefore have implications for patients’ knowledge of MS and risk knowledge. Further, only published studies were included in the review, potentially excluding further relevant findings.

Patients in Europe or North America were overrepresented, with two studies reporting on patients within South America [37] and Asia [9]. This limits generalisability of findings to geographic regions where knowledge characteristics may differ and reveals opportunities for more cross-cultural research into how patients’ disease and risk knowledge can be measured and characterised.

Patients were recruited from varied sources, including academic research centres, clinics, online advertisements and MS newsletters. It is possible those recruited from different settings vary in baseline characteristics, such as exposure to disease information. Any possible implication recruitment method has for patients’ disease knowledge requires further exploration in research. Further, MS-related cognitive impairments were seldom measured in studies of MS knowledge and MS risk knowledge despite having significant influence on patients’ appraisal of health risks and decision making [52].

A further consideration, which was not investigated in the present review, relates to the increased provision and availability of disease-related information via the internet and its bearing on patients’ knowledge and care quality. MS patients have been identified as more likely to seek information from the internet than those with other long-term conditions [53], with greater use having been associated with younger age and higher socioeconomic status [54]. However, such information can be subject to inaccuracies, which may have implications for patients’ knowledge, with patients having reported concerns about quality of information on the internet [53]. The way in which the quality and availability of clinical and technical information via the internet has shaped patients’ knowledge, the overall nature of their care, and their engagement with treatment, warrants further study.

### Future directions

While outstanding knowledge needs have been identified, these can be advanced through interventions [21, 23]. Improving knowledge may have implications for making decisions about and engaging in treatment. Identifying patients with knowledge needs through validated assessments may inform tailored interventions. Further, included studies measuring MS knowledge have not applied criteria to define gradations in knowledge, while few studies applied numeric thresholds to measure changes in knowledge following educational interventions. Reliable evaluation of educational programmes could be increased through using knowledge measures with consistent criteria to categorise patients’ knowledge and are revised in tandem with the evolving MS landscape [55]. Relatedly, it remains to be investigated following the present review to what extent patients’ knowledge characteristics and needs evolve over time according to scientific advances in understanding MS, the availability of related information, and an individual’s accrued lived experience with MS. Development in cross-culturally validated and standardised measures of MS knowledge and MS risk knowledge may also be fruitful next steps for research and clinical practice. Obtaining culturally normative measures of disease and risk perception may help ascertain where there are variations in MS patients’ knowledge.

### Conclusion

Studies have highlighted suboptimal MS knowledge and MS risk knowledge in patients. Sampling and measurement differences preclude direct comparisons between studies. Higher levels of education were correlated with higher levels of knowledge. Interventions can enhance knowledge, with important implications for treatment decision making and adherence. Refinements in how disease knowledge is measured may allow more definitive conclusions regarding patients’ disease knowledge.

### Supplementary information

Below is the link to the electronic supplementary material.

Supplementary file1 (PDF 153 KB)

Supplementary file2 (PDF 153 KB)

## Data Availability

All data in this review have been extracted from cited articles.
