# Supplementary material for: A systematic review to explore patients’ MS knowledge and MS risk knowledge
Source: Neurol Sci. 2024 May 3;45(9):4185–95. doi: 10.1007/s10072-024-07541-5 (PMC11306520; doi:10.1007/s10072-024-07541-5)
Supplement: Supplementary file 1 — Supplementary file1 (PDF 153 KB) [file 10072_2024_7541_MOESM1_ESM.pdf]

Supplementary Information 1 - Table S1

Study Demographics

First Author	Sample Size (IG; CG)	Age (years) Mean (SD)[†]	Female	Country of recruitment/ Ethnicity	Education	Employment	MS Subtype	Years Since Diagnosis Mean (SD)
Abulaban et al. [9]	200	18-30: 42.5%<br>30-45: 48.5%<br>45-55: 9%	66%	Saudi Arabia 100%/ (NS[‡])	< High school 9.5%<br>High school 26%<br>Diploma 12.5%<br>Bachelor 51.5%<br>PhD 0.5%	Student 7.5%<br>Teacher 16%<br>Health care provider 7%<br>Engineer 4%<br>Military 3.5%<br>Unemployed 49.5%<br>Retired 3%<br>Other 9.5%	-	NS (NS)
Bichuetti et al. [37]	96	39.3 (11.9)	-	Brazil 100%/ (NS)	Mean years of schooling: 12.6 (SD = 3.0)	-	RRMS 100%	9.1 (6.8)
Bruce et al. [26]	290	49.3 (11.6)	80.3%	USA 100%/<br>Caucasian 86.9%<br>African 8.6%	Bachelor's degree 31%<br>Graduate-professional 21%	Full time employment 33%<br>Unemployed 32.1%	RRMS 74.8%<br>SPMS 16.9%<br>PPMS 6.9%<br>PRMS 1.4%	12 (NS)
Bruce et al. [27]	208	46 (10)	83.2%	USA 100%/<br>Caucasian 87%<br>African American 7.7%<br>Hispanic 2.4%<br>Asian 0.5%<br>NS 2.4%	College education 13.9%<br>Bachelor's degree 29.3%<br>Advanced degrees 21.2%<br>Reported some college 20.7%<br>High school education or less 14.9%	-	RRMS 100%	11 (8)

First Author	Sample Size (IG; CG)	Age (years) Mean (SD)[†]	Female	Country of recruitment/ Ethnicity	Education	Employment	MS Subtype	Years Since Diagnosis Mean (SD)
Feicke et al. [31]	64 (33; 31)	IG: 41.9 (11.7)	IG: 87.1%	Germany 100%/ (NS)	IG: Lower secondary school 19.4%<br>Secondary school 35.5%<br>Highschool 45.2%	IG: Full time 41.9%<br>Half time 19.4%<br>Unemployed 38.7%	IG: RRMS 38.7%<br>SPMS 6.5%<br>PPMS 3.2%<br>Unclear 45.2%	IG: 1 (1.1)
		CG: 37.1 (7.8)	CG: 69.7%		CG: Lower secondary school 21.2%<br>Secondary school 57.6%<br>Highschool 21.2%	CG: Full time 45.5%<br>Half time 21.2%<br>Unemployed 33.3%	CG: RRMS 69.7%<br>PPMS 3%<br>Unclear 27.3%	CG: 1.6 (1.5)
Giordano et al. [11]	102	35.2 (9.7)	67.6%	Italy 100%/ (NS)	Primary 28.4%<br>Secondary 54.9%<br>College/University 16.7%	Full or part time 73.5%<br>Homemaker 11.8%<br>Student 10.8%<br>Retired 0.0%<br>Unemployed 3.9%	RRMS 91.2%<br>PPMS or SPMS 8.8%	-
Giordano et al. [28]	986	38.6	77%	Germany 19%/ (NS)<br>Italy 9%/ (NS)<br>Serbia 11%/ (NS)<br>Spain 28%/ (NS)<br>Netherlands 14%/ (NS)<br>Turkey 20%/ (NS)	Graduate:<br>Germany 49%<br>Italy 44%<br>Serbia 57%<br>Spain 53%<br>Netherlands 42%<br>Turkey 50%	-	RRMS 100%	7.8 (NS)

First Author	Sample Size (IG; CG)	Age (years) Mean (SD)[†]	Female	Country of recruitment/ Ethnicity	Education	Employment	MS Subtype	Years Since Diagnosis Mean (SD)
Heesen et al. [15]	169	44 (11)	62.7%	Germany 100%/ (NS)	> 12 years 40.8%	-	RRMS 44%<br>PPMS 44%<br>Disease course <1 year 12%	7.7 (6.9)
Heesen et al. [16]	34 (Pilot)	39.6 (Range 27–48)	76%	Germany 100%/ (NS)	≥ Secondary school 94%<br>Primary school 6%	-	Early RRMS[¶] 12%<br>RRMS 59%<br>SPMS 27%<br>Unclear 2%	5.1 (NS)
	192[§] (93;99) (RCT)	36.6 (Range 18-70)	74%	Germany 100%/ (NS)	University degree 23%<br>Secondary school 52%<br>Primary school 25%		Early RRMS 83%<br>Unclear 16%	1.3 (NS)
Heesen et al. [10]	708	39.8 (10.2)	26.3%	Germany 100%/ (NS)	≥ 12 years 52%<br>10–11 years 35%<br>≤ 9 years 13%	-	RRMS 68%<br>SPMS 11%<br>PPMS 6%<br>Unclear 7%	7.1 (6.7)
Heesen et al. [38]	99	38.1 (9.2)	53.5%	Germany 100%/ (NS)	-	-	RRMS 100%	7.7 (5.8)
Hofmann et al. [39]	575	50.3 (9.8)	64.5%	Germany 100%/ (NS)	-	-	RRMS 8.5%<br>SPMS 44.9%<br>PPMS 13.2%<br>Other 0.7%<br>Unclear 32.7%	14.3 (median) 1.5-39 (range)
Jarmolowicz et al. [29]	244	49 (12)	79.4%	USA 100%/ White 86%	College graduate 65.4%	Full time 32.9%	RRMS 74.7%<br>PPMS 17%<br>PRMS 1.2%	-

First Author	Sample Size (IG; CG)	Age (years) Mean (SD)[†]	Female	Country of recruitment/ Ethnicity	Education	Employment	MS Subtype	Years Since Diagnosis Mean (SD)
Köpke et al. [34]	192 (93; 99)	IG: 36.5 (10.3)	IG: 74%	Germany 100%/ (NS)	IG: University degree 27% >12 years 57%	IG: Full time 48% At least half time 60%	IG: RRMS 73% Unclear 15%	IG: 1.4 (0.9)
		CG: 36.7 (10.3).	CG: 75%		CG: University degree 20% >12 years 47%	CG: Full time 40% At least half time 67%	CG: RRMS 65% Unclear 18%	CG: 1.2 (0.8)
Köpke et al. [35]	156 (75; 81)	IG: 42.2 (8.9)	IG: 82.7%	Germany 100%/ (NS)	IG: >12 years 43.2%	IG: At least half time employment 70.4%	IG: RRMS 66.2% SPMS 9.5% PPMS 4% Unclear 17.6%	IG: 7 (7)
		CG: 42.5 (10.3).	CG: 67.9%		CG: >12 years 40%	CG: At least half time employment 63%	CG: RRMS 70% SPMS 8.8% PPMS 12.5% Unclear 5%	CG: 9 (8.7)
Prunty et al. [32]	139 (78; 61)	IG: 32 (3.8)	100%	Australia 100%/ (NS)	-	-	IG: RRMS 75% SPMS 2% PPMS 1% Unknown 6%	-
		CG: 31.1 (5.6)					CG: RRMS 63% Unknown 5%	

First Author	Sample Size (IG; CG)	Age (years) Mean (SD)†	Female	Country of recruitment/ Ethnicity	Education	Employment	MS Subtype	Years Since Diagnosis Mean (SD)
Rahn et al. [36]	73 (38; 35)	IG: 38.8 (9)	IG: 68%	Germany 100%/ (NS)	IG: Higher education 51.1%	IG: At least part time employment 74.3%	RRMS 100%	IG: 41.4 (56.5) months
		CG: 36.2 (11)	CG: 80%		CG: Higher education 69%	CG: At least part time employment 88.9%		CG: 56.5 (79.5) months
Skinner et al. [33]	81	44.8	66.1%	Canada 100%/ (NS)	High school graduate 22.6%<br>Attended high school 4.8%<br>Attended post-secondary education 11.3%<br>University graduate 46.8%<br>Post-graduate degree 12.9%	Employed 67.7%<br>Unemployed 6.5%<br>On disability 12.9%<br>Other 12.9%	RRMS 22.6%<br>PPMS 6.5%<br>SPMS 4.8%<br>Unconfirmed 32.2%<br>‘I don’t know’ 33.9%	-

Note: IG = Intervention group; CG = Control group. †Unless otherwise reported. ‡Not Specified. §Analysed data from Köpke et al.’s [34] study. ¶< 2 years from diagnosis of RRMS.
