# Supplementary material for: A systematic review to explore patients’ MS knowledge and MS risk knowledge
Source: Neurol Sci. 2024 May 3;45(9):4185–95. doi: 10.1007/s10072-024-07541-5 (PMC11306520; doi:10.1007/s10072-024-07541-5)
Supplement: Supplementary file 2 — Supplementary file2 (PDF 153 KB) [file 10072_2024_7541_MOESM2_ESM.pdf]

Supplementary Information 2 - Table S2

Study Results

First Author	Recruitment Method	Study Design	Sample Size (*n*) (IG; CG)	Follow-up Period (Years)†	MS Knowledge Measure	MS Risk Knowledge Measure	Results
Abulaban et al. [9]	Participants recruited via support from MS patient support group in Saudi Arabia.	Survey	200	-	MSKQ‡	-	The mean MS knowledge score was 13.6 (SD = 3.6). Scores ranged from 4 to 21.
Bichuetti et al. [37]	Participants attending appointments at two MS clinics in Brazil.	Survey	96	-	-	Knowledge of developing progressive multifocal leukoencephalopathy when taking natalizumab measured by 5 multiple choice answers, ranging from 1:100,000 to 1:200.	Regarding the chance of developing progressive multifocal leukoencephalopathy when taking natalizumab:<br>15% (*n* = 14) reported a 1:100,000 chance,<br>13 % (*n* = 12) a 1:10,000 chance,<br>39% (*n* = 37) a 1:1,000 chance,<br>8% (*n* = 8) a 1:500 chance,<br>26% (*n* = 25) a 1:200 chance.

First Author	Recruitment Method	Study Design	Sample Size (*n*) (IG; CG)	Follow-up Period (Years)†	MS Knowledge Measure	MS Risk Knowledge Measure	Results
Bruce et al. [26]	Participants recruited via approach in MS clinics, online adverts, mailed letters to patients, and adverts in *Mid-America MS Society Newsletter*.	Survey	290	-	MSKQ§	-	The mean MS knowledge score was 17.4 (SD = 3.4). Scores ranged from 3 to 24.
Bruce et al. [27]	Via MS clinic and advert in *Mid-America MS Society Newsletter*.	Survey	208	-	MSKQ§	-	The mean MS knowledge score was 17.5 (SD = 3.4). Scores ranged from 3 to 24.
Feicke et al. [31]	Recruited via invitation from facilitators of the 'S.MS' programme or by neurologists.	Quasi-experimental	64 (33; 31)	0.5	Bespoke knowledge questionnaire to assess contents of the training programme - 14 questions with the Yes, No and Don't Know options.	-	At baseline, participants in the IG provided correct responses to 76.9% of the MS knowledge questions. Participants in the CG: provided correct answers to 76.4% of the MS knowledge questions.

First Author	Recruitment Method	Study Design	Sample Size (*n*) (IG; CG)	Follow-up Period (Years)[†]	MS Knowledge Measure	MS Risk Knowledge Measure	Results
Giordano et al. [11]	MS patients recruited from two Italian centres for Phase I testing. Newly diagnosed patients from five Italian centres recruited for Phase II testing (validation sample II) who were included in the SIMS-Trial.	Survey	102 (Valid-ation sample II)	-	MSKQ[§]	-	The median MS knowledge score was 17. Scores ranged from 3 to 24.

First Author	Recruitment Method	Study Design	Sample Size (n) (IG; CG)	Follow-up Period (Years)[†]	MS Knowledge Measure	MS Risk Knowledge Measure	Results
Giordano et al. [28]	Depending on country, via charity homepages in Germany and Spain, via national MS society mailing lists in Italy and Netherlands, via personal approach in MS clinics in Serbia and Turkey.	Survey	986	-	MSKQ[§]	RIKNO 2.0[¶]	The mean MS knowledge score was 19.3 (SD = 3.2) in patients completing both the RIKNO 2.0 and MSKQ (n = 298). The mean MS risk knowledge score across the whole sample was 8.7 (SD = 3.5).
Heesen et al. [15]	Randomly selected from a German MS Outpatient Clinic.	Survey	169	-	Subjective perceived level of knowledge[††] of therapeutic decisions rated on a visual analogue scale.	MSK[‡‡] (also referred to as the RIKNO)	Participants rated their subjective perceived level of knowledge as 63%. The mean MSK score was 6.4 (SD = 2.4).

First Author	Recruitment Method	Study Design	Sample Size (*n*) (IG; CG)	Follow-up Period (Years)[†]	MS Knowledge Measure	MS Risk Knowledge Measure	Results
Heesen et al. [16]	Random selection from a German MS day hospital database.	Cohort	34 (Pilot)	-	-	RIKNO 1.0[§§]	The mean MS risk knowledge score was 9.8 (SD = 3.2) for pilot study patients. Scores ranged from range 0 to 16.
	Correlational analyses conducted on data obtained from participants in the PEPADIP RCT Köpke et al. [34].		192 (93; 99)			RIKNO 1.0	The mean MS risk knowledge score was 10.2 (SD = 2.8) for patients in the PEPAPIP RCT. Scores ranged from 0 to 16.
Heesen et al. [10]	Patients with RRMS recruited via advertisements within MS outpatient clinics.	Survey	708	-	MSKQ[§]	RIKNO 2.0	The mean MS knowledge score was 20.1 (SD = 2.6). The mean MS risk knowledge score was 8.9 (SD = 3.6).

First Author	Recruitment Method	Study Design	Sample Size (*n*) (IG; CG)	Follow-up Period (Years)[†]	MS Knowledge Measure	MS Risk Knowledge Measure	Results
Heesen et al. [38]	Patients undergoing or planning to undergo treatment with NAT selected by neurologists within participating MS centres in Germany.	Prospective Observational Cohort	99	1	-	Knowledge of PML[¶¶] assessed through seven multiple choice questions on its related risk factors. Scores range from 0-7 and a mean score is calculated.	Patients' knowledge of PML risk stratification was lower than neurologists' (47.8% correct; SD = 27.5; Mann-Whitney U = 6.5, $p < .001$).
Hofmann et al. [39]	Hospital and private practices in Germany who had treated at least five patients with mitoxantrone between 1991 and 2010 carried out database searches of eligible patients and invited to participate.	Retro-spective Cohort	575	-	-	Bespoke measures of baseline MS knowledge and risk perception for developing leukaemia.	40% (n = 117) correctly selected a risk of 8:1000 from 8:10, 8:100, 8:1,000 and 8:10,000 for leukaemia. 58% (n = 165) incorrectly selected a risk of 8:10,000.

First Author	Recruitment Method	Study Design	Sample Size (*n*) (IG; CG)	Follow-up Period (Years)[†]	MS Knowledge Measure	MS Risk Knowledge Measure	Results
Jarmolowicz et al. [29]	Participants recruited from a large MS speciality clinic and via adverts in the *Mid-America MS Society Newsletter*.	Survey	244	-	MSKQ[§]	-	Mean MS knowledge scores were 17.1 (SD = 3.5). Scores ranged from 3 to 24.
Köpke et al. [34]	Participants recruited from 6 German university-based MS clinics using flyers. Staff also informed eligible patients attending the MS clinics.	RCT	192 (93; 99)	1	-	RIKNO 1.0	Mean baseline MS risk knowledge were 10.6 (SD = 2.6) for participants in the IG, and 9.4 (SD = 2.9) for those in the CG.
Köpke et al. [35]	Recruited by clinic staff within 3 rehabilitation centres in Germany.	CCT	139 (68; 71)	0.5	-	RIKNO 1.0	Mean baseline MS risk knowledge scores for those in the IG was 6.1 (SD = 2.8) and 6.5 (SD = 2.5) for those in the CG.

First Author	Recruitment Method	Study Design	Sample Size (*n*) (IG; CG)	Follow-up Period (Years)[†]	MS Knowledge Measure	MS Risk Knowledge Measure	Results
Prunty et al. [32]	Members of Australian MS societies willing to participate in research contacted via posted letter.	RCT	139 (78; 61)	2 weeks	MS knowledge through a bespoke knowledge questionnaire.[†††]	-	Mean baseline MS knowledge scores for those in the IG was 4.1 (SD = 1.8) and 4.2 (SD = 1.9) for those in the CG.
Rahn et al. [36]	Participants from two German MS university centres approached by physicians and nurses.	RCT	73 (38; 35)	0.5	-	RIKNO 1.0	Mean baseline MS risk knowledge scores for those who were in the IG was 8.3 (SD = 3.4) and 8.1 (SD = 3.1) for those in the CG.

First Author	Recruitment Method	Study Design	Sample Size (n) (IG; CG)	Follow-up Period (Years)[†]	MS Knowledge Measure	MS Risk Knowledge Measure	Results
Skinner et al. [33]	Patients due to be seen by a genetic counsellor between at a Canadian MS clinic invited to participate before their genetic counselling appointment.	Cohort	81	-	Prior to genetic counselling the 'PRE survey' was completed and included questions about perceived under-standing of the causes of MS.	-	At baseline 43.5 % (n = 27) rated their understanding of the causes of MS as "average," 17.8 % (n = 11) "greater than average" and 38.7 % (n = 24) "less than average."

Note: IG = Intervention group; CG = Control group. [†]Unless otherwise reported. [‡]Multiple Sclerosis Knowledge Questionnaire - maximum score = 23. [§]Multiple Sclerosis Knowledge Questionnaire - maximum score = 25. [¶]Risk Knowledge in RRMS 2.0 - maximum score = 21. [††]A perceived level of knowledge at 100% represents maximal knowledge. [‡‡]MS Risk Knowledge Questionnaire - maximum score = 19. [§§]Risk Knowledge in RRMS 1.0 - maximum score = 19. [¶¶]Knowledge of progressive multifocal leukoencephalopathy (PML) was reported as percentages of correct responses. [†††]Maximum knowledge scores were 10 with higher scores denoting increased knowledge.
